# Correction to: Deep learning for improving PET/CT attenuation correction by elastic registration of anatomical data

**DOI:** 10.1007/s00259-023-06199-z

**Published:** 2023-03-25

**Authors:** Joshua Schaefferkoetter, Vijay Shah, Charles Hayden, John O. Prior, Sven Zuehlsdorff

**Affiliations:** 1 Siemens Medical Solutions USA, Inc, 810 Innovation Drive, Knoxville, TN 37932 USA; 2 Department of Nuclear and Molecular Imaging, Lausanne University Hospital, Lausanne, Switzerland; 3 University of Lausanne, Lausanne, Switzerland

**Correction to: European Journal of Nuclear Medicine and Molecular Imaging**

**https://doi.org/10.1007/s00259-023-06181-9**

The article Deep learning for improving PET/CT attenuation correction by elastic registration of anatomical data, written by Joshua Schaefferkoetter, Vijay Shah, Charles Hayden, John O. Prior, and Sven Zuehlsdorff, was originally published online on the publisher’s internet portal on March 8, 2023 with Open Access under a Creative Commons Attribution (CC BY) license 4.0. With the author’s/authors' decision to cancel Open Access the copyright of the article changed on March 11, 2023 to © The Author(s), under exclusive licence to Springer-Verlag GmbH Germany, part of Springer Nature 2023 with all rights reserved.

The original article has been corrected.

